# FEV Maintains Homing and Expansion by Activating ITGA4 Transcription in Primary and Relapsed AML

**DOI:** 10.3389/fonc.2022.890346

**Published:** 2022-07-07

**Authors:** Jubin Zhang, Lijuan Qi, Tanzhen Wang, Jingnan An, Biqi Zhou, Yanglan Fang, Yujie Liu, Meng Shan, Dengli Hong, Depei Wu, Yang Xu, Tianhui Liu

**Affiliations:** ^1^ National Clinical Research Center for Hematologic Diseases, Jiangsu Institute of Hematology, The First Affiliated Hospital of Soochow University, Suzhou, China; ^2^ Institute of Blood and Marrow Transplantation, Collaborative Innovation Center of Hematology, Soochow University, Suzhou, China; ^3^ Key Laboratory of Cell Differentiation and Apoptosis of Ministry of Education, Department of Pathophysiology, Shanghai Jiao Tong University School of Medicine (SJTU-SM), Shanghai, China

**Keywords:** FEV, AML (acute myeloid leukemia), ITGA4, homing, expansion

## Abstract

Acute myeloid leukemia (AML) is an aggressive hematological malignancy that recurs in approximately 50% of cases. Elevated homing and uncontrolled expansion are characteristics of AML cells. Here, we identified that Fifth Ewing Variant (FEV) regulates the homing and expansion of AML cells. We found that *FEV* was re-expressed in 30% of primary AML samples and in almost all relapsed AML samples, and *FEV* expression levels were significantly higher in relapsed samples compared to primary samples. Interference of *FEV* expression in AML cell lines delayed leukemic progression and suppressed homing and proliferation. Moreover, FEV directly activated integrin subunit alpha 4 (*ITGA4*) transcription in a dose-dependent manner. Inhibition of integrin α4 activity with natalizumab (NZM) reduced the migration and colony-forming abilities of blasts and leukemic-initiating cells (LICs) in both primary and relapsed AML. Thus, our study suggested that FEV maintains the homing and expansion of AML cells by activating *ITGA4* transcription and that targeting *ITGA4* inhibits the colony-forming and migration capacities of blasts and LICs. Thus, these findings suggested that the *FEV*-*ITGA4* axis may be a therapeutic target for both primary and relapsed AML.

## Introduction

Acute myeloid leukemia (AML) is an aggressive hematological malignancy in which immature cells accumulate and expand uncontrollably. Nearly 50% of patients relapse after induction chemotherapy (1, 2). Although new approaches have improved the prognosis of some patients, the majority of primary and relapsed AML patients still lack effective treatment (1–4). Therefore, there is an urgent need for novel targets or drugs to improve the outcomes of these patients.

Enhanced homing and migration abilities are features of AML cells, which rapidly home to bone marrow (BM) and hijack the normal hematopoietic niche to aid extensive expansion of leukemic initiating cells (LICs) (5–7). Cell-to-cell or cell-to-matrix interactions mediated by C-X-C Motif Chemokine Receptor 4 (CXCR4)- C-X-C Motif Chemokine Ligand 12 (CXCL12), integrins and CD44 signaling pathways have been reported to contribute to the homing of leukemic cells to the BM microenvironment (5–11). However, the upstream mechanism that activates the pathways of VLA-4 remains unknown. Here, we identified that fifth Ewing variant (FEV) regulates integrin signaling.

FEV (also known as PET1 in mammals) is an E26 transformation-specific transcription factor (12, 13). *FEV* was initially reported to regulate the synthesis of 5-hydroxytryptamine (5-HT), and *FEV* deficiency results in the differentiation arrest of the majority of 5-HT–producing neurons and a 70–80% decrease in 5-HT (14, 15). In recent years, FEV has been reported to be a functional regulator in the generation and self-renewal of embryo hematopoietic stem cells (HSCs) (13, 16). In our previous work, we reported that *FEV* is silenced in normal adult hematopoiesis and re-expressed in leukemias of prenatal origin, and *FEV* deficiency significantly impairs the leukemia-propagating capacity of LICs in AML patient-derived xenograft mice (17). However, the mechanism of how FEV modulates the reconstitution of LICs remains unknown.

In the present study, we demonstrated that *FEV* was re-expressed in 30% of primary and in almost all relapsed AML samples with high expression levels, and we found that FEV maintains the homing and expansion abilities of AML cells by directly activating integrin subunit alpha 4 (*ITGA4*) transcription. Integrin α4 blockade inhibited the migration and colony-forming abilities of blasts and LICs in both primary and relapsed AML. Our results demonstrated the role of the *FEV*-*ITGA4* axis in homing and expansion maintenance, providing a potential therapeutic target for primary and relapsed AML.

## Materials and Methods

### Human Samples

Human bone marrow (BM) aspirates of patients with leukemia from 2018 to 2020 were obtained from the First Affiliated Hospital of Soochow University, and the studies were approved by the Medical Ethical Committees of the hospital in accordance with the Declaration of Helsinki protocol. All human participants signed a written informed consent form. In total, 69 AML samples containing 16 relapsed AML samples were included. The detailed information for the clinical samples is shown in **Supplementary Tables 1–3**. Mononuclear cells (MNCs) from the samples were obtained using Ficoll (Sigma–Aldrich, St. Louis, MO, USA) gradient centrifugation and frozen in liquid nitrogen.

### Xenograft Model

NOD-SCID mice (aged 6–8 weeks) were purchased from Shanghai SLAC Laboratory Animal Co., Ltd., Shanghai, China. The mice were bred under pathogen-free conditions at the Laboratory Animal Center of Soochow University. All animal experiments were performed in accordance with the protocols approved by the Experimental Animal Ethical Committee at Soochow University. The NOD-SCID mice were irradiated with 2.0 Gy of X-rays and subsequently treated with CD122 antibody at a dose of 200 μg per mouse *via* intraperitoneal injection. The mice were injected with genetically modified MV4-11 cells at a dose of 1 × 10^5^ through the caudal vein.

### Real-Time Quantitative Polymerase Chain Reaction

Total RNA was extracted using a TRIzol reagent in accordance with the manufacturer’s instructions and reversely transcribed. RT-qPCR was also performed using TB Green Premix Ex Taq II (Takara Bio, Otsu, Japan) in accordance with the manufacturer’s instructions. All experiments were performed in triplicate with ABI QuantStudio 3 Real-time PCR System (Applied Biosystems, MA, USA). The primer sequences are summarised in **Supplementary Table 4**. Differences in cDNA input were normalized to the *ACTB* expression levels. *FEV* positivity was defined in accordance with a previous report (17).

### Chromatin Immunoprecipitation

ChIP assays were performed using the Magna ChIP A/G kit (Merck Millipore, Billerica, MA, USA) in accordance with the manufacturer’s instructions. MV4-11 cells with ectopically expressed flag-FEV were cross-linked in 1% formaldehyde and then sonicated to create soluble chromatin. Antibodies against flag (Sigma–Aldrich, St. Louis, MO, USA) were added to precipitate the DNA fragments. The recovered DNA was amplified using PCR or quantitative PCR.

### Homing Assay

Homing assays were performed as previously reported (18). In this assay, 2 × 10^6^ transduced MV4-11 cells were injected into sublethally irradiated NOD-SCID mice by the caudal vein. 16 hours later, bone marrow (BM) cells of the recipients were stained with human CD45-PC7 and analysed using a flow cytometer (ACEA Biosciences, California, USA). All antibodies were obtained from BD Bioscience.

### RNA Sequencing

MV4-11 cells transduced with NC or shFEV were cultured for 3 days and sorted by BD FACS Melody (BD Biosciences, San Jose, CA, USA). The cells were collected and high-throughput RNA sequencing (RNA-seq) was performed by Illumina HiSeq 2500 (Illumina, San Diego, CA) at CapitalBio Corporation (Beijing, China). Genes with a *P* value < 0.05 and fold changes ≥ 1.5 were recognized as differentially expressed genes between the two samples (Dataset 1). Pathway analysis (q value ≤ 0.001) of these differentially expressed genes was conducted using KOBAS 2.0, and the analysed results are provided in **Supplementary Table 5**.

### Statistical Analysis

The Mann–Whitney U test was used to estimate the differences in *FEV* expression. The definition of *FEV* positivity was based on a previous report (17). The *FEV* positivity was analyzed using the chi-squared test. The survival of xenograft mice was assessed using the Kaplan–Meier method and the log-rank test. Statistical analysis was conducted using SPSS 25.0 software (SPSS Inc., Chicago, USA) and GraphPad Prism software (version 8.4.1, GraphPad Software, San Diego, CA).

## Results

### FEV Is Re-Expressed in Primary and Relapsed AML With High Expression Levels

**Figure 1:**
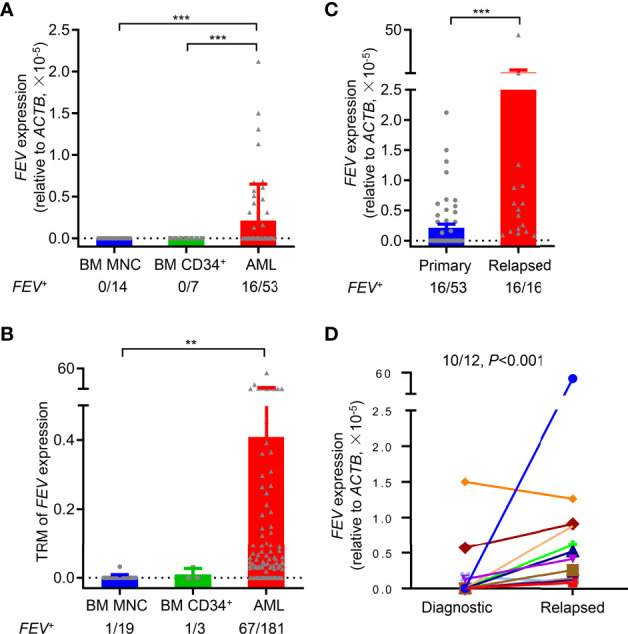
*FEV* is highly expressed in primary and relapsed AML. **(A)** *FEV* mRNA expression levels in primary AML samples, and BM MNCs and CD34^+^ cells from healthy donors were used as normal controls. **(B)** *FEV* mRNA expression levels were analyzed using the data from the Vizome database. **(C)** Comparison of *FEV* mRNA levels in samples from primary and relapsed AML. The proportion of *FEV* ^+^ samples and relative mRNA level are indicated. Positivity for *FEV* expression was identified as previously described (17). **(D)** *FEV* mRNA expression levels in paired diagnostic and relapsed samples. *FEV* mRNA was significantly higher in 91.7% (11/12) of relapsed samples than in the diagnostic samples. **P < 0.01 and ***P < 0.001 **(A–C)**, Mann–Whitney U test; **(D)**, Wilcoxon test. All data are presented as the mean ± SD.

To validate *FEV* expression in AML, BM aspirates from 53 primary AML patients were examined using reverse transcription-polymerase chain reaction (RT–PCR), and BM mononuclear cells (MNCs) and CD34^+^ cells from healthy donors were also examined as controls. According to the definition of *FEV* positivity from a previous report (17), *FEV* was negative in all normal BM MNCs (14/14) and CD34^+^ cells (7/7), and 30.2% (16/53) of primary AML samples were *FEV* positive (**Figure 1A**, **Supplementary Table 1**). Next, *FEV* expression was validated using the Vizome database (19) (www.vizome.org), which indicated that 37.0% (67/181) of primary AML samples were *FEV* positive (**Figure 1B**).

To explore whether *FEV* is preferentially expressed in AML patients, the clinical characteristics of the patients who were diagnosed and treated in our center were summarized. *FEV* was no preferentially expressed in age, sex, FAB subtypes, genetics (except for CEBPA) and cytogenetics. However, statistical analysis showed a worse risk category in *FEV* ^+^ patients compared with the *FEV* ^-^ group (*P*=0.0017), according to the guideline of ELN risk stratification. Also, a significantly lower rate of complete remission was observed after two courses of induction chemotherapy in *FEV* ^+^ group (12.5% vs. 94.6%, *P*<0.001, **Supplementary Table 2**). In addition, the *FEV* ^+^ patients had unfavorable 2-year overall survival (OS) and relapse-free survival (RFS) than the *FEV* ^-^ patients (OS: 46.63% vs. 69.17%, *P*=0.020; RFS: 25.00% vs. 55.39%, *P*=0.030) (**Supplementary Figures 1A, B**). Therefore, we considered that *FEV* was preferentially expressed in aggressive and treatment-refractory subsets of AML.

BM samples from relapsed AML patients were also analyzed to further study *FEV* expression at different stages of AML progression, and their clinical characteristics were shown in **Supplementary Table 3**. All of the relapsed samples (16/16) were *FEV* positive, and the mRNA levels of *FEV* were significantly higher in relapsed samples than in primary samples (**Figure 1C**). However, 55.6% (5/9) of relapsed samples were *FEV* positive, and no significant difference in *FEV* expression was observed between primary and relapsed samples using data in the Vizome database, which may due to the small sample size (**Supplementary Figure 1C**). We next confirmed *FEV* expression in patients with paired diagnostic and relapsed samples. *FEV* expression was significantly higher in 83.3% (10/12) of relapsed samples than in the diagnostic samples (**Figure 1D**). These results indicated that *FEV* was re-expressed in 30% of primary AML samples and in almost all relapsed AML samples, and high *FEV* expression levels were determined in relapsed samples, suggesting that *FEV* may play a role in AML progression.

### FEV Deficiency Inhibits the Colony-Forming Ability and Proliferation of AML Cells *in Vitro*

FEV expression in AML leukemic cell lines was examined by RT–PCR and immunoblotting (**Supplementary Figures 2A, B**), and *FEV* positively expression cell lines, MV4-11, THP-1 and KG-1 cells, were used to investigate the role of *FEV* in AML progression. *FEV* knockdown was achieved using lentiviral vector-driven interference shRNAs (shFEVs), and the most effective shRNAs (**Supplementary Figure 2C**) were selected as previously reported (17).

**Figure 2:**
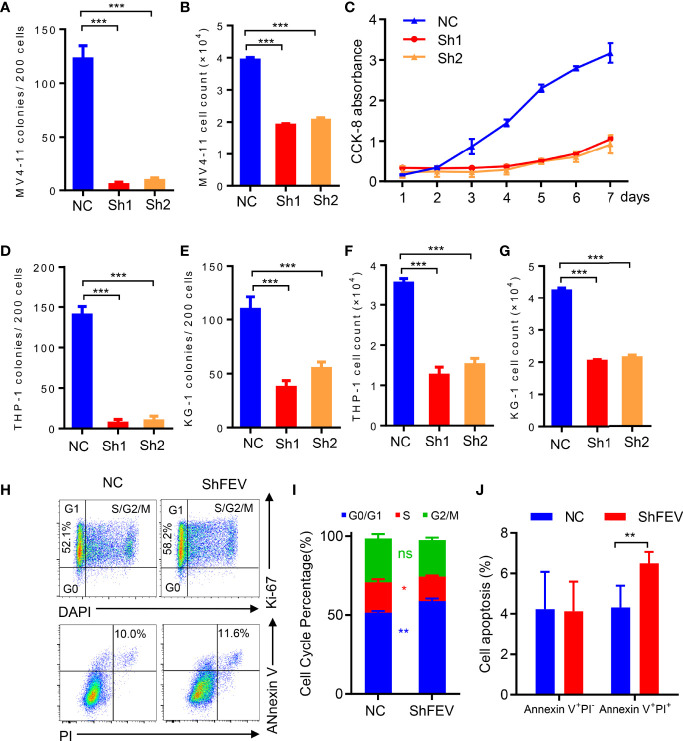
*FEV* deficiency inhibits the colony-forming ability and proliferation of AML cells *in vitro*. **(A, D, E)** Number of colonies formed by sorted MV4-11 **(A)**, THP-1 **(D)** and KG-1 **(E)** cells transduced with nonsilencing control (NC), *FEV* shRNA1 (sh1) or shRNA2 (sh2) after 7 days. **(B, F, G)** Cell counts in sorted MV4-11 **(B)**, THP-1 **(F)** and KG-1 **(G)** cells transduced with NC, sh1 or sh2 after 2 days of culture. **(C)** CCK-8 assay showing the effect of shFEV on proliferation over a period of 7 days for MV4-11 cells. **(H)** Representative flow cytometry images of the cell cycle and apoptosis of MV4-11 cells transduced with NC or shFEV at 2 days. **(I, J)** Statistical analysis of the percentage of cells in G1 phase **(I)** and Annexin V^+^ cells **(J)** in the NC and shFEV groups. The results are representative of at least three independent experiments. *P < 0.05, **P < 0.01 and ***P < 0.001 (Student’s t test). All data are presented as the mean ± SD.

MV4-11 cells transduced with nonsilencing control (NC), *FEV* shRNA1 (sh1) or shRNA2 (sh2) were flow-sorted according to GFP positivity, and colony-forming cell (CFC) assay and cell proliferation assay were conducted. We found that *FEV* knockdown markedly reduced CFCs (**Figure 2A**). The cell count in the shFEV group was decreased after 2 days of culture (**Figure 2B**), which was confirmed by proliferation detection after 7 days of culture (**Figure 2C**). Reduced CFCs (**Figures 2D, E**) and inhibited proliferation (**Figures 2F, G**) were also observed in THP-1 or KG-1 cells transduced with shFEVs. The cell cycle status and apoptosis of AML cells were also evaluated. A slight increase in the frequency of G1 phase and a mild decrease in the frequency of S phase were observed in FEV-deficient cells, but no significant difference was found between the NC and shFEV (sh1) groups in terms of total Annexin V^+^ cells although a slight increase in the proportion of late apoptosis was observed. (**Figures 2H** **–J**), suggesting that *FEV* knockdown leads to proliferation inhibition with G1 arrest.

### FEV Deficiency Inhibits the Homing Ability of AML Cells *in Vivo*

**Figure 3:**
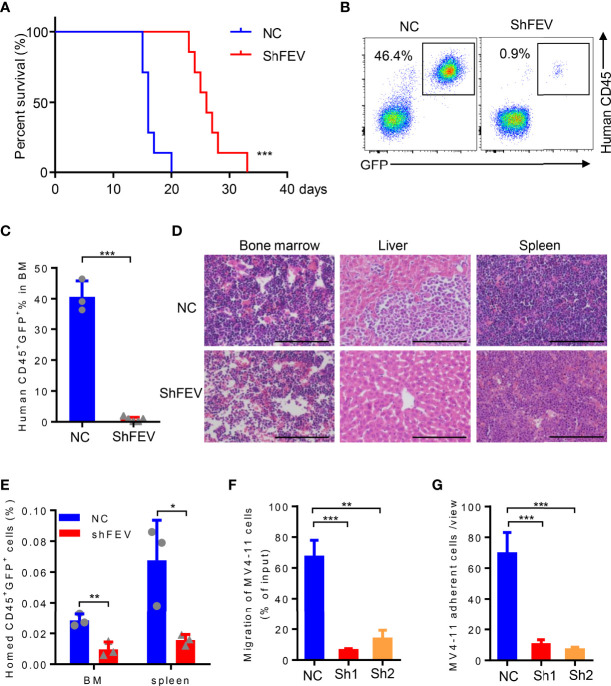
*FEV* deficiency inhibits the homing ability of AML cells *in vivo*. **(A)** Kaplan–Meier plot of disease-free survival of mice that received MV4-11 cells transduced with NC or shFEV (n=7). **(B, C)** Representative flow cytometry plot **(B)** and statistical analysis **(C)** of the percentage of engrafted cells in BM from mice that received NC (n=3) or shFEV MV4-11 cells after 15 days (n=5). **(D)** HE-stained sections (size bars = 50 μm) of BM, spleen and liver of mice that received NC or shFEV MV4-11 cells after 15 days. **(E)** Statistical analysis of the frequency of NC or shFEV cells homed to the BM and spleen at 16 h(n=3). **(F)** Frequency of migrating cells in the NC, sh1 and sh2 groups, which was normalized to the input control. **(G)** The average count of adherent MV4-11 cells in 10 random views. The results are representative of at least three independent experiments. *P < 0.05, **P < 0.01 and ***P < 0.001 (Student’s t test). All data are presented as the mean ± SD.

MV4-11 cells transduced with NC or shFEV (sh1, GFP^+^) were transplanted into immunodeficient NOD-SCID mice treated with CD122 antibody (NS122 mice) (17, 20, 21). The mice that received *FEV* knockdown cells had extensively prolonged survival time compared to their NC counterparts (**Figure 3A**). *FEV* deficiency significantly reduced the engraftment of AML cells in the BM (**Figures 3B** **–D**), which was consistent with previous findings in leukemia with prenatal initiation (17). *FEV* knockdown also reduced the infiltration of leukemic cells in the spleen and liver (**Figure 3D** and **Supplementary Figure 3A–C**), indicating the impaired homing and migration abilities of the leukemic cells.

Given that enhanced homing and migration are the characteristics of leukemic cells, losing them may result in delayed leukemia progression (5–11). Therefore, the homing ability of leukemic cells after *FEV* knockdown was examined. Transduced MV4-11 cells were injected into sublethally irradiated NOD/SCID mice followed by analysis of human CD45^+^GFP^+^ cells in the spleen and BM 16 hours after the injection. The AML cells that homed to the BM or spleen were significantly decreased in the shFEV group (**Figure 3E** and **Supplementary Figure 3D**), indicating that *FEV* knockdown impairs the homing ability. *In vitro* transwell and adhesion assays supported that *FEV* knockdown decreased the frequency of migrated cells (**Figure 3F** and **Supplementary Figure 3E**) and those adherent to fibronectin (**Figure 3G** and **Supplementary Figure 3F**). Furthermore, the migration and adhesion abilities of *FEV*-deficient THP-1 or KG-1 cells were also inhibited (**Supplementary Figures 3G–J**).

### Integrin Signaling Is Inhibited in FEV-Deficient AML Cells

**Figure 4:**
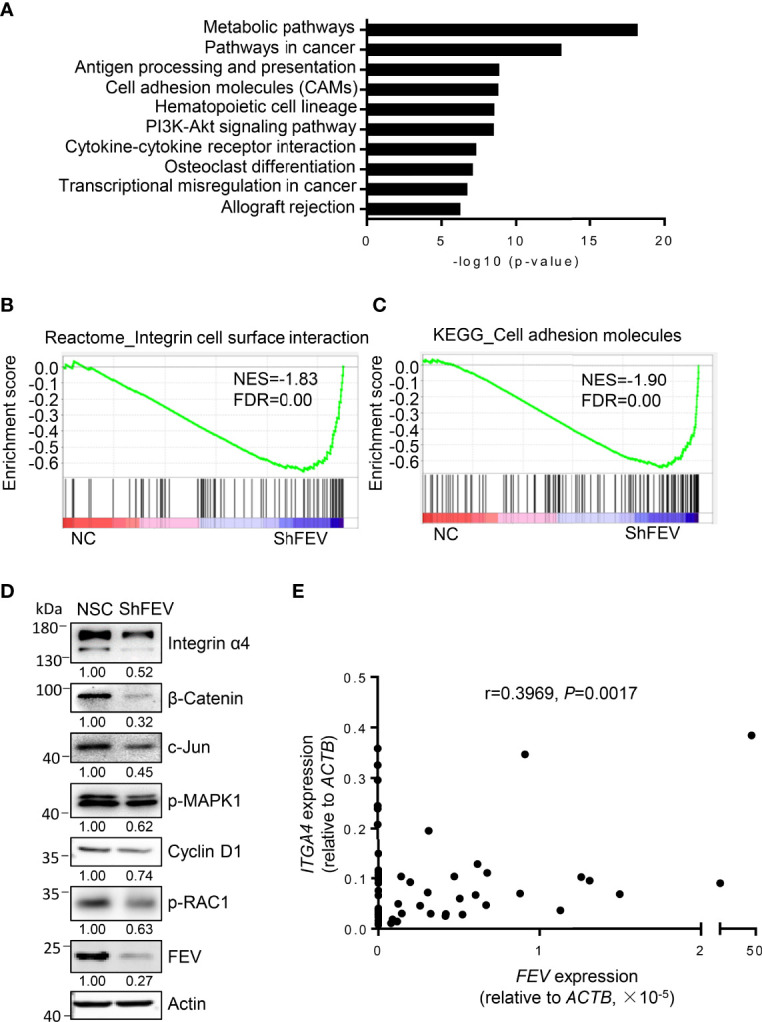
Integrin signaling is inhibited in *FEV*-deficient AML cells. **(A)** KEGG analysis highlighted the alteration of signaling pathways in MV4-11 cells transduced with NC or shFEV. **(B, C)** GSEA of selected signatures encoding the gene sets of integrin cell surface interaction **(B)** and cell adhesion molecules **(C)**, which were differentially expressed between NC and shFEV cells. **(D)** Immunoblotting analysis of protein expression in the integrin signaling pathway. Relative protein levels were quantified using ImageJ software, and the numbers refer mean signals for three independent experiments. **(E)** Correlation analysis between *FEV* and *ITGA4* expression in AML samples.

MV4-11 cells transduced with NC or shFEV (sh1) were flow-sorted and subjected to gene transcriptome examination to investigate the underlying mechanisms. Kyoto Encyclopedia of Genes and Genomes (KEGG) analysis highlighted an intensive expression alteration of genes involved in cell adhesion molecules (CAMs), transcriptional misregulation in cancer and the PI3K-Akt signaling pathway (**Figure 4A**). Gene set enrichment analysis (GSEA) indicated that the *FEV*-deficient cells had a decreased enrichment of integrin cell surface interactions (**Figure 4B**) and cell adhesion molecules (**Figure 4C**). The mRNA and protein expression levels of genes related to the pathways were validated *via* RT–qPCR and western blot analyses. Key regulators of integrin signaling (integrin α4, phosphorylated RAC1, phosphorylated MAPK1, c-Jun and β-catenin) were downregulated (**Figure 4D** and **Supplementary Figures 4A–D**), indicating that the integrin signaling pathway may be downstream of FEV.

Given that integrins are key regulators of cell adhesion, migration and proliferation (22–25) and that *ITGA4* is highly expressed in leukemic cells (26), *ITGA4* may be a potential target of FEV. The correlation between *FEV* and *ITGA4* expression in AML samples was analyzed, and a significant correlation between *FEV* and *ITGA4* (**Figure 4E**) was observed. These results indicated that *ITGA4* may be the target of FEV.

### FEV Regulates AML Progression by Directly Activating ITGA4 Transcription

To study whether ITGA4 mediates FEV functions in AML progression, *ITGA4* expression was interfered with by shRNAs (sh-ITGA4). Consistent with *FEV* knockdown, the number of CFCs was reduced (**Supplementary Figure 5A**), the proliferation of AML cells was inhibited (**Supplementary Figure 5B**) and the frequency of cells in G1 phase was increased (**Supplementary Figure 5C**) in the sh-ITGA4 group. Notably, cyclin D1 expression was decreased (**Supplementary Figure 5D**) in sh-ITGA4 cells, suggesting that ITGA4 may regulate the expansion of AML cells. In mice that received MV4-11 cells with *ITGA4* interference, the survival time was extended (**Supplementary Figure 5E**), the reconstitution of leukemic cells was decreased (**Supplementary Figure 5F**), the infiltration of leukemic cells was decreased (**Supplementary Figure 5G**) and the frequency of cells homed to the BM or spleen was reduced (**Supplementary Figure 5H**). *In vitro* transwell and adhesion assays also exhibited a decrease in the proportion of migrated (**Supplementary Figure 5I**) and adhered (**Supplementary Figure 5J**) MV4-11 cells with *ITGA4* interference.

**Figure 5:**
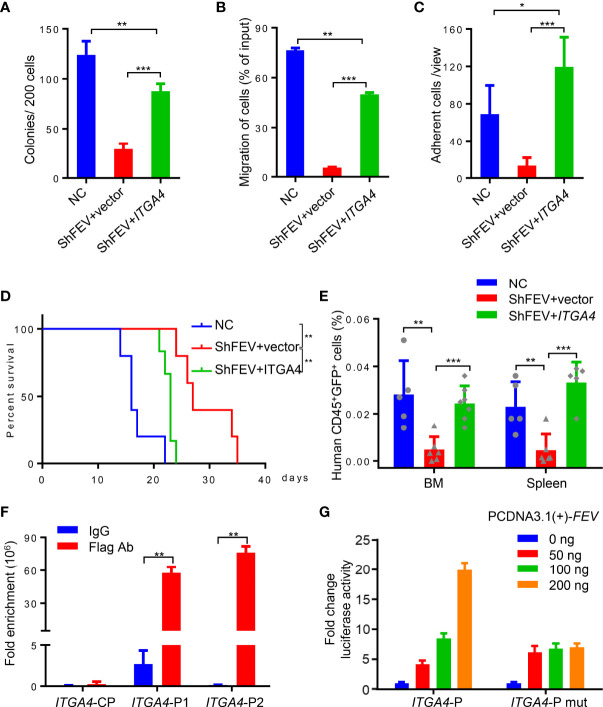
FEV regulates AML progression by directly activating *ITGA4* transcription. **(A)** Number of colonies formed by NC and shFEV cells transduced with empty vector (shFEV+vector) or ectopically expressed *ITGA4* (shFEV+*ITGA4*) after 7 days. **(B)** Frequency of migrated cells in the NC, shFEV+vector and shFEV+*ITGA4* groups. **(C)** Average count of adherent cells in 10 random views. **(D)** Kaplan–Meier plot of disease-free survival of mice that received MV4-11 cells transduced with NC (n=5), shFEV+vector (n=5) or shFEV+*ITGA4* (n=6). **(E)** Frequency of cells homed to the BM and spleen following intravenous injection in the NC (n=5), shFEV+vector (n=6) and shFEV+*ITGA4* groups at 16 h (BM n=7; spleen n=5). **(F)** ChIP-qPCR analysis of FEV enrichment on the *ITGA4* promoter. CP indicates the control sites without the FEV-binding site. P1 and P2 indicate the regions with conserved FEV-binding sites. **(G)** Dual luciferase reporter assays using *ITGA4* promoter constructs that contain conserved (*ITGA4*-P) or mutated (*ITGA4*-P mut) FEV-binding sites cotransfected with the pCDNA3.1-*FEV* plasmid. The results are representative of at least three independent experiments. *P < 0.05, **P < 0.01 and ***P < 0.001 (Student’s t test). All data are presented as the mean ± SD.

*ITGA4* was then ectopically expressed in FEV-knockdown MV4-11 cells, which were then subjected to *in vitro* and *in vivo* assays. Compared to the shFEV group, the number of colonies was significantly increased, the proliferation was significantly increased and G1 arrest was alleviated in the *ITGA4*-expressing shFEV group (**Figure 5A** and **Supplementary Figures 6A, B**). Migration and adhesion inhibition was also partly alleviated in *ITGA4*-expressing shFEV cells (**Figures 5B** **, C**). The mice that received *ITGA4*-expressing shFEV cells showed a significantly reduced survival time, which was comparable to that of their NC counterparts (**Figure 5D**). After *ITGA4* expression, the frequency of infiltrated leukemic cells in organs was increased (**Supplementary Figures 6C, D**), and the homing arrest in shFEV cells to the BM and spleen was fully reversed (**Figure 5E**). Therefore, these findings indicated that *ITGA4* is essential to *FEV* functions in expansion and homing of AML cells.

Given that FEV is a transcription factor, the present study investigated whether FEV regulates the transcription of *ITGA4*. Two regions containing potential FEV-binding sites in the *ITGA4* promotor were found by JASPAR (**Supplementary Figures 6E, F**). Chromatin immunoprecipitation (ChIP)-PCR assays with MV4-11 leukemic cells demonstrated that FEV binds to both regions (**Supplementary Figure 6G**). The amount of DNA fragments bound to the FEV protein was quantified by qPCR, and the results revealed that FEV preferred to bind to the P2 region (**Figure 5F**). The core binding sites in both regions were then mutated to TCTTCCCT to exclude nonspecific binding. A luciferase reporter assay in 293T cells was conducted using the promoter constructs of *ITGA4* containing wild-type (*ITGA4*-P) or mutant FEV-binding sites (*ITGA4*-P mut). The results suggested that FEV positively regulated wild-type *ITGA4* expression in a dose-dependent manner but not mutant *ITGA4* (**Figure 5G**), indicating that *ITGA4* is the direct target of FEV.

### Blocking ITGA4 Activity Reduces the Colony-Forming, Migration and Adhesion Abilities of Blasts and LICs in Primary and Relapsed AML Patients

Because *ITGA4* was identified as a downstream target of FEV in AML cells, we next investigated whether *ITGA4* can be a target in the treatment of AML. Natalizumab (NZM), a therapeutic antibody approved by the U.S. Food and Drug Administration (FDA) for multiple sclerosis (27) and Crohn’s disease (28), was used to block the activity of integrin α4. Consistent with FEV knockdown, MV4-11 cells treated with NZM exhibited a decrease in the count of CFCs (**Supplementary Figure 7A**), migrated cells (**Supplementary Figure 7B**) and adhesion cells (**Supplementary Figure 7C**). After treatment with NZM, the proliferation of MV4-11 cells was inhibited, and an increased frequency of G1 phase cells was also found (**Supplementary Figures 7D, E**).

The samples of primary AML were thawed and treated with NZM and subjected to colony-forming, migration and adhesion assays. Thirteen primary AML samples were divided into *FEV* ^-^ and *FEV* ^+^ groups according to *FEV* positivity. In *FEV* ^+^ samples, NZM treatment reduced the number CFCs in the blasts (**Supplementary Figure 8A**) and inhibited the ability to migrate and adhere to fibronectin (**Supplementary Figures 8B, C**). However, no significant difference between IgG and NZM treatment was observed in the *FEV* ^-^ group, indicating that *ITGA4* is a specific target of FEV.

**Figure 6:**
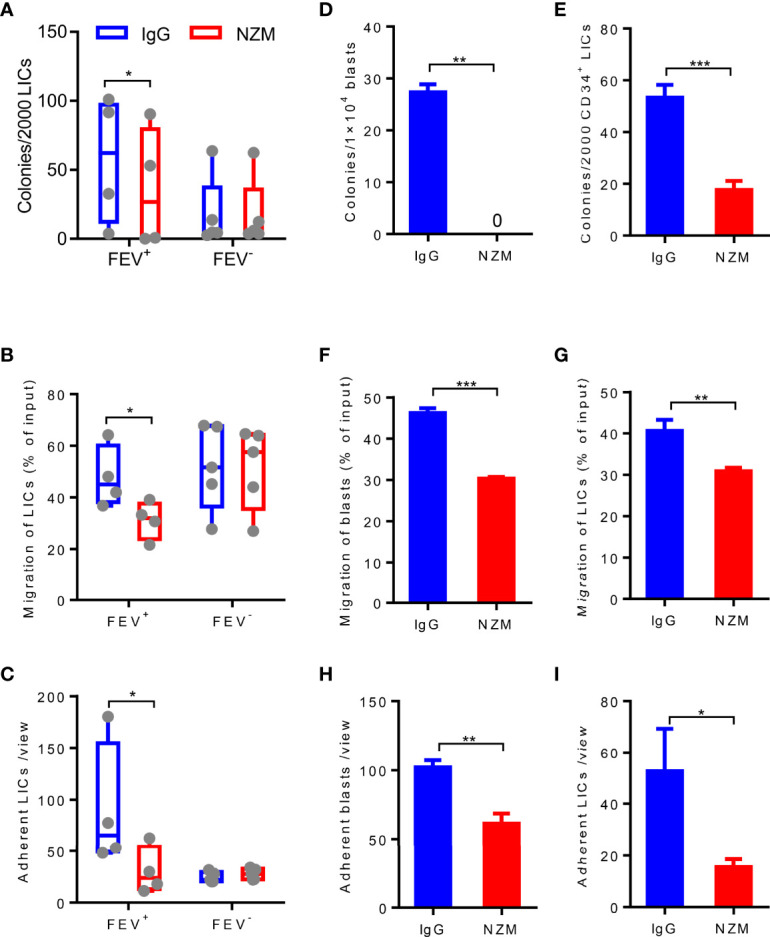
Blocking integrin α4 activity reduces the colony-forming, migration and adhesion abilities of blasts and LICs in primary and relapsed AML patients. **(A)** Number of colonies formed by LICs sorted from primary AML patients after IgG4 or NZM treatment after 14 days. **(B)** Frequency of migrated LICs from primary AML patients after 2 days of IgG4 or NZM treatment. **(C)** Average count of adherent LICs from primary AML patients per view. **(D, E)** Number of colonies formed by blasts **(D)** and LICs **(E)** from relapsed AML patients after IgG4 or NZM treatment. **(F, G)** Frequency of migrated blasts **(F)** and LICs **(G)** from relapsed AML patients after 2 days of IgG4 or NZM treatment. **(H, I)** Average count of adherent blasts **(H)** and LICs **(I)** per view from relapsed AML patients after IgG4 or NZM treatment. *P < 0.05, *P < 0.05, **P < 0.01 and ***P < 0.001 **(A–C)**, paired t test; D-I, Student’s t test. All data are presented as the mean ± SD.

Given that LICs are the main cause of relapse (7, 8, 29) and previous evidence has reported that the CD34^+^CD38^−^CD123^+^ putative LICs maintains high expression of integrin α4β1 (VLA-4) (30), we next determined whether LICs are eliminated by NZM treatment. CD34^+^CD38^-^ LICs were sorted from 10 primary AML patients and treated with NZM. Consistent with blasts, LICs showed a reduced colony-forming capacity (**Figure 6A**), and the frequency of LICs that migrated or adhered to fibronectin was decreased after NZM treatment in the *FEV* ^+^ group (**Figures 6B, C**). Moreover, no significant difference was observed in the *FEV* ^-^ group.

To determine whether NZM affects relapsed AML samples, blasts and LICs from relapsed AML patients were treated with NZM. The abilities of relapsed blasts or LICs to form colonies (**Figures 6D, E**), migrate (**Figures 6F, G**) and adhere (**Figures 6H, I**) were significantly inhibited after NZM treatment. These results suggested that ITGA4 may be a potential therapeutic target for relapsed AML.

In conclusion, *FEV* was re-expressed in 30% of primary and in almost all relapsed AML samples with high expression levels. FEV is functionally required for AML progression by regulating the homing and expansion of AML cells. *ITGA4* is a direct target of FEV, and blockade of *FEV-ITGA4*-mediated homing and expansion may be a novel approach for primary and relapsed AML therapy.

## Discussion

Approximately 40–45% of AML patients will achieve remission with current standard chemotherapy, but disease recurrence will appear in nearly 50% of these patients (1, 2). The prognosis of patients with relapsed AML is poor with no more than 10% overall survival in 3 years (1–4). Although new approaches, such as epigenetic drugs, leukemic antigen antibodies, inhibitors (for FLT3, IDH1, MDM2 and BCL2) and CAR-T therapy, have improved the outcome of some patients (2, 31, 32), a large number of primary or relapsed patients still lack effective treatment.

Here, we identified FEV as a regulator of AML progression and identified that the *FEV*-*ITGA4* axis is involved in the homing and expansion of AML cells, which may be a potential therapeutic target for primary and relapsed AML.

In previous work, we reported that *FEV* is silenced in adult hematopoiesis and re-expressed in leukemias of prenatal origin, such as infants, children and young adults (<40 years); we also demonstrated that FEV is essential for leukemia propagation of LICs (17), but the mechanism is not clear. In the present study, we confirmed that *FEV* was re-expressed in approximately 30% of primary AML samples (our hospital, 30.2% FEV^+^; and Vizome database, 37.0% FEV^+^). The expression of *FEV* was associated with worse risk category, CR rate after 2 course of induction chemotherapy and prognosis. Most importantly, we observed that *FEV* was expressed in almost all relapsed AML samples, and the expression levels of *FEV* were higher in relapsed samples than in primary samples. We further demonstrated that *FEV* was required for AML progression. *FEV* deficiency markedly reduced the homing and expansion abilities of AML cells. These findings revealed the mechanism underlying the function of FEV in the propagation of LICs.

Leukemic cells are retained in the BM microenvironment with elevated homing and migration abilities, which are mediated by cell-to-cell or cell-to-matrix interactions. CXCR4-CXCL12 signaling, VLA4 signaling and CD44 signaling have been reported to contribute to the homing of leukemic cells to the BM microenvironment (5–11). However, the upstream signaling pathway remains unknown. In the present study, we found a significant correlation between *FEV* and *ITGA4* (α4 subunit of VLA4) expression in AML samples, and ectopic *ITGA4* expression elevated homing and expansion of shFEV MV4-11 cells and accelerated AML progression. ChIP and luciferase assays demonstrated that *ITGA4* was a downstream target of FEV, which activated *ITGA4* transcription by directly binding to the promoter in a dose-dependent manner. Thus, we identified a novel *FEV-ITGA4* axis for homing and migration.

VLA4 is highly expressed in leukemic blasts and LICs (30), and it mediates the attachment of blasts and LICs to the ECM or stromal cells (5–11). High expression of VLA4 is associated with adverse outcomes in AML (26), chronic lymphocytic leukemia (33–35) and B-cell precursor acute lymphoblastic leukemia (36). Disruption of the adhesion of blasts within the microenvironment by a VLA-4 antibody or small molecular inhibitors sensitizes drug-resistant acute lymphoblastic leukemia (37–39) and AML (40, 41) to chemotherapy. In the present study, we showed that integrin α4 blockade with NZM resulted in reduced colony-forming, adhesion and migration abilities of blasts and LICs. NZM treatment also decreased the CFCs as well as the adhesion and migration of relapsed AML cells, although the account of colonies was less than that from fresh samples. Thus, these findings suggested that *ITGA4* may be a potential target for both primary and relapsed AML. In agreement with Hsieh *et al.* (41, 42), blasts and LICs were not eradicated completely after NZM treatment; therefore, combination with cytotoxic chemotherapy agents or other targeted drugs may be a better approach.

In summary, our data provides mechanistic insight into the role of the *FEV*-*ITGA4* axis in the progression of AML and the treatment application of NZM in primary and relapsed AML. Therefore, *ITGA4* may be considered a therapeutic target for both primary and relapsed AML.

## Data Availability Statement

The datasets presented in this study can be found in online repositories. The names of the repository/repositories and accession number(s) can be found below: https://www.ncbi.nlm.nih.gov/geo/, GSE166621.

## Ethics Statement

The studies involving human participants were reviewed and approved by the Medical Ethical Committees of the First Affiliated Hospital of Soochow University. The patients/participants provided their written informed consent to participate in this study. The animal study was reviewed and approved by the Experimental Animal Ethical Committee at Soochow University.

## Author Contributions

JZ performed the experiments and wrote the manuscript. LQ and TW performed the experiments and analyzed the results. BZ and YF established the xenograft model. YL and MS collected the patient samples and clinical information. JA, DH, YX and DW discussed the data and contributed to the writing of the manuscript. TL supervised the project, designed the experiments and wrote the paper. All authors contributed to the article and approved the submitted version.

## Funding

This work was supported by grants from the National Key Natural Science Foundation of China (81730003), the Excellent Youth Science Fund of Jiangsu Province (BK20211553), the Natural Science Foundation of China (81700139 and 81870120), the National Science and Technology Major Project (2017ZX09304021), the National Key R&D Program of China (2019YFC0840604 and 2017YFA0104502), the Key R&D Program of Jiangsu Province (BE2019798 and BE2019655), the Natural Science Fund of Jiangsu Province (BK20170360), the Priority Academic Program Development of Jiangsu Higher Education Institutions (PAPD), the Jiangsu Medical Outstanding Talents Project (JCRCA2016002) and the Jiangsu Provincial Key Medical Center (YXZXA2016002).

## Conflict of Interest

The authors declare that the research was conducted in the absence of any commercial or financial relationships that could be construed as a potential conflict of interest.

## Publisher’s Note

All claims expressed in this article are solely those of the authors and do not necessarily represent those of their affiliated organizations, or those of the publisher, the editors and the reviewers. Any product that may be evaluated in this article, or claim that may be made by its manufacturer, is not guaranteed or endorsed by the publisher.
