# Supplementary material for: FEV Maintains Homing and Expansion by Activating ITGA4 Transcription in Primary and Relapsed AML
Source: Front Oncol. 2022 Jul 7;12:890346. doi: 10.3389/fonc.2022.890346 (PMC9300928; doi:10.3389/fonc.2022.890346)
Supplement: Supplementary file 1 [file DataSheet_1.pdf]

Supplementary Material

Contents

Supplementary Methods
Supplementary Figure Legends 1-8
Supplementary Figures 1-8
Supplementary Tables 1-5

Supplementary Methods

Lentivirus production

FEV (TRCN0000015295 and TRCN0000015294, Sigma–Aldrich) and *ITGA4* interference oligonucleotides (TRCN0000029654, Sigma–Aldrich) were cloned into pLko.1 vector. Lentiviruses were packaged as previously described (1). Cell lines were infected with lentivirus at an MOI of 20 for 18 h. Transduced cells were sorted by BD Melody (BD Biosciences, San Jose, CA, USA), and these sorted cells were either transplanted into NOD-SCID mice or used for *in vitro* assays.

Cell culture

The human leukemic cell lines were cultured in accordance with the ATCC's instruction. The MV4-11, THP-1 and KG-1 cells were cultured in RPMI medium (Hyclone, UT, USA) supplemented with 10% FBS (Biological Industries, Kibbutz, Israel) and 1% penicillin/streptomycin in humidified 37 °C incubator containing 5% CO_2. The blasts and LICs from primary or relapsed AML patients were cultured in SFEM medium (StemCell Technologies, Vancouver, BC, Canada) supplemented with 1% antibiotic–antimycotic (GIBCO BRL, Grand Island, NY, USA), 20 ng/ml human TPO, 20 ng/ml human SCF, and 20 ng/ml human FLT3-ligand (PeproTech, Rocky Hill, NJ, USA). The MV4-11 cells, blasts or LICs from patients were treated with 10 ug/ml natalizumab (NZM) or IgG4 (Merck Millipore, Billerica, MA, USA) for 1–3 days, followed by *in-vitro* assays.

Vizome Database Analysis

We downloaded the RNA sequencing and clinical data from Vizome database, and 181 bone barrow samples from primary AML cases, 9 relapsed AML cases, 19 MNCs and 3 CD34 cells from healthy donors were included in the analysis.

Colony-forming cell (CFC) assay

For cell lines, CFC assays were performed in a semi-solid methylcellulose medium by using MethoCult H4100 (StemCell Technologies, Vancouver, BC, Canada). The cells were plated in 24-well plate containing methylcellulose. The colonies were counted under a microscope after 7–10 days of culture. For primary leukemia cells, CFC assays were performed with 1×10^4 blasts or 2000 LICs using MethoCult H4435 (StemCell Technologies, Vancouver, BC, Canada) in a 24-well plate, and colonies were counted after 13–14 days of culture.

Luciferase Assays

293T cells were transiently cotransfected with the pcDNA3.1-*FEV* plasmid and pGL-*ITGA4*-promoter or pGL-*ITGA4* mutation promoter plasmid using Lipofectamine 2000 (Thermo Fisher Scientific, Waltham, MA, USA). Luciferase activity was quantified 48 h after transfection using dual-luciferase reporter assay systems (Promega, Madison, WI, USA). Firefly luciferase activity was normalized to the Renilla activity following

the manufacturer's instructions.

Transwell assay

Transwell assays were performed as previously reported (2). MV4-11 cells or leukemic cells from patients with AML patients were seeded into 8 μM pore membrane inserts (BD Biosciences, New Jersey, USA) precoated with 20 μg/mL fibronectin (Sigma–Aldrich, St. Louis, MO, USA). A total of 3×10^5 cells were added on the upper chamber. Another 3×10^5 cells were added directly into the lower chambers as input. After 12 h of incubation, the cells in the lower chamber were collected and 1×10^5 MV4-11 cells with GFP expression were added. The mixture was detected using the flow cytometer.

Adhesion assay

Adhesion assays were performed as previously reported (3-5). Firstly, a 96-well plate was precoated with 20 μg/ml of fibronectin (Sigma–Aldrich, St. Louis, MO, USA) and blocked with 1% BSA solution for 1 h at 37 °C. Next, 5×10^4 cells per well were seeded. After 1 h of incubation, the cells were rinsed and then fixed with methanol (Sinopharm Chemical Reagent, Shanghai, China) for 15 min at room temperature. The attached cells were finally stained with Wright's Giemsa dye (Solarbio, Beijing, China) and counted under an inverted microscope (Nikon, Tokyo, Japan).

Cell Counting Kit-8 (CCK-8) Assay

Cells were cultured in 96-wells plate for 2000 cells per well, and cell viability was quantitated by the Cell Counting Kit-8 (CCK-8; Bimake, Houston, Texas, USA) for 7 days. Cells were added with CCK-8 and incubated for 4 h at 37°C, and then measured at the absorbance of 450 nm wavelength according to the manufacturer's instructions.

Cell cycle

The MV4-11 cells were collected 1–3 days after being flow-sorted or treated with NZM or IgG4, followed by fixing and permeabilization with Cytofix/Cytoperm Buffer (BD Pharmingen, San Diego, USA) for 30 min on ice. The cells were washed with BD Perm/Wash Buffer and stained with Ki-67-APC (BioLegend, San Diego, CA, USA) for 30 min on ice. Then, cells were stained with DAPI (4′,6-diamidino-2-phenylindole; Beyotime Biotechnology, Shanghai, China) at a concentration of 500 ng/ml for 20 min on room temperature in the dark and then washed with PBS. The stained cells were analysed using a flow cytometer (ACEA Biosciences, California, USA).

Apoptosis

The MV4-11 cells were resuspended in binding buffer and incubated with Annexin V-APC at room temperature for 15 min before staining with propidium iodide solution (BioLegend, San Diego, CA, USA) at 4 °C in the dark. The stained cells were analysed using a flow cytometer (ACEA Biosciences, California, USA).

Engraftment analysis

The BM cells of the recipients were flushed down with Iscove's modified Dulbecco's

medium (Gibco, CA, USA) supplemented with 1% BSA (Sigma–Aldrich, St. Louis, USA). The cells were treated with ACK solution (150 mM NH_4Cl, 1 mM $KHCO_3$, and 0.1 mM EDTA) at room temperature for 10 min to lyse red blood cells. The cells were immediately washed and then resuspended in PBS supplemented with 1% BSA for antibody staining at 4 °C for 30 min in the dark. The cells were stained with human CD45-PC7 for engraftment. The stained cells were analysed using a flow cytometer (ACEA Biosciences, California, USA). All antibodies were obtained from BD Bioscience.

Western Blot

The cells were lysed using cold RIPA (Beyotime Biotechnology, Shanghai, China) with 1 mM PMSF, 100X Protease Inhibitor Cocktail II, and Phosphatase Inhibitor Cocktail (Cell Signaling Technology, Danvers, MA, USA). Lysate was centrifuged at 12,000 × g for 5 min at 4 °C, and the supernatant was loaded as protein samples. Following 8%–12% SDS-PAGE separation, the proteins were electrophoresed and transferred to nitrocellulose membranes (Merck Millipore, Billerica, MA, USA). After blocking with 5% non-fat milk for 1 h, the nitrocellulose membranes were incubated with antibodies for FEV (Thermo Fisher Scientific, Waltham, MA, USA), actin, integrin α4, cyclin D1, β-catenin, phosphorylated-MAPK1, RAC1 and phosphorylated-RAC1 (Cell Signaling Technology, Danvers, MA, USA) overnight. The membranes were washed in TBST three times, each for 5 min, and incubated for 2 h with anti-rabbit secondary antibody or anti-mouse secondary antibody (Sigma–Aldrich, St. Louis, MO, USA). After extensive washing, the membranes were detected using a chemiluminescent HRP substrate (Thermo Fisher Scientific, Waltham, MA, USA). Relative protein levels were quantified using ImageJ software.

References

1. Liu TH, Tang YJ, Huang Y, Wang L, Guo XL, Mi JQ, et al. Expression of the Fetal Hematopoiesis Regulator Fev Indicates Leukemias of Prenatal Origin. *Leukemia* (2017) 31(5):1079-86. doi: 10.1038/leu.2016.313.
2. Voermans C, van Heese WP, de Jong I, Gerritsen WR, van Der Schoot CE. Migratory Behavior of Leukemic Cells from Acute Myeloid Leukemia Patients. *Leukemia* (2002) 16(4):650-7. doi: 10.1038/sj.leu.2402431.
3. Kortlepel K, Bendall LJ, Gottlieb DJ. Human Acute Myeloid Leukaemia Cells Express Adhesion Proteins and Bind to Bone Marrow Fibroblast Monolayers and Extracellular Matrix Proteins. *Leukemia* (1993) 7(8):1174-9. Epub 1993/08/01.
4. Cancelas JA. Adhesion, Migration, and Homing of Murine Hematopoietic Stem Cells and Progenitors. *Methods Mol Biol* (2011) 750:187-96.
5. Singh V, Erb U, Zoller M. Cooperativity of Cd44 and Cd49d in Leukemia Cell Homing, Migration, and Survival Offers a Means for Therapeutic Attack. *J Immunol* (2013) 191(10):5304-16. doi: 10.4049/jimmunol.1301543.

Supplementary Figure Legends

Supplementary Figure 1. The prognisis of FEV$^+$ and FEV$^-$ patients. (A-B) Overall survival **(A)** and relapse-free survival **(B)** of *FEV*$^+$ and *FEV*$^-$ patients. **(C)** *FEV* mRNA expression levels in primary and relapsed AML samples using the data from the Vizome database.

Supplementary Figure 2. FEV expression in leukemic cell lines. (A) FEV expression in leukemic cell lines detected by RT-PCR. PC, cDNA from cord blood CD34$^+$ cells was used as positive control. NC, negative control, using water as template. **(B)** Protein levels of FEV in leukemic cells were validated by western blot. **(C)** FEV expression in MV4-11 cells transduced with nonsilencing control (NC), FEV shRNA1 (sh1) or shRNA2 (sh2).

Supplementary Figure 3. FEV deficiency inhibits the homing ability of AML cells. (A-C) Representative flow cytometry plot (**A**) and statistical analysis of the percentage of engrafted cells in spleen (**B**) and liver (**C**) from mice that received NC (n=3) or shFEV MV4-11 cells (n=5) after 15 days. **(D)** Representative flow cytometry plot of cells homed to BM and spleen stained with human CD45 antibody and GFP (n=3) at 16 h. **(E)** Representative plot of migrating cells in the NC, sh1 and sh2 groups, which was normalized to the input control. **(F)** Representative plot of the adherent cells in the NC, sh1 and sh2 groups. **(G, H)** Frequency of migrating cells in sorted THP-1 (**G**) and KG-1 (**H**) cells, which was normalized to the input control. **(I, J)** The average count of adherent THP-1 (**I**) and KG-1 (**J**) cells in 10 random views. The results are representative of at least three independent experiments. $^{**}P < 0.01$ and $^{***}P < 0.001$ (Student's t test). All data are presented as the mean ± SD.

Supplementary Figure 4. Integrin signaling is inhibited in FEV-deficient AML cells. (A-D) Relative mRNA level of ITGA4 (**A**), CRK (**B**), CCNE2 (**C**), CCND1 (**D**) genes in NC or shFEV MV4-11 cells. The results are representative of at least three independent experiments. $^{**}P < 0.01$ and $^{***}P < 0.001$ (Student's t test). All data are presented as the mean ± SD.

Supplementary Figure 5. ITGA4 is required for the homing and expansion of AML cells. (A) Number of colonies formed by sorted MV4-11 transduced with NC or sh-ITGA4 after 7 days. **(B)** CCK-8 assay showing the effect of sh-ITGA4 on proliferation over a period of 7 days for MV4-11 cells. **(C)** Statistical analysis of the percentage of cells in G1 phase in the NC and sh-ITGA4 groups. **(D)** Immunoblotting analysis of integrin α4, cyclin D1, RAC and p-RAC expression in NC and sh-ITGA4 cells. **(E)** Kaplan–Meier plot of disease-free survival of mice that received MV4-11 cells transduced with NC or sh-ITGA4 (n=5). **(F)** Engraftment of human CD45$^+$GFP$^+$ cells in BM, spleen and liver from mice that received NC or sh-ITGA4 MV4-11 cells after 15 days (n=4). **(G)** HE-stained sections (size bars = 50 μm) of BM, spleen and liver of mice that received NC or sh-ITGA4 MV4-11 cells after 15 days. **(H)** Frequency of human CD45$^+$GFP$^+$ cells homed to the BM and spleen in the NC (BM n=3; spleen n=5)

or sh-ITGA4 group at 16 h (n=3). **(I)** Frequency of migrating MV4-11 cells transduced with NC or sh-ITGA4. **(J)** Average count of adherent MV4-11 cells transduced with NC or sh-ITGA4 in 10 random views. The results are representative of at least three independent experiments. *P < 0.05, **P < 0.01 and ***P < 0.001 (Student's t test). All data are presented as the mean ± SD.

Supplementary Figure 6. FEV regulates AML progression by directly activating ITGA4 transcription. (A) CCK-8 assay of NC and shFEV cells transduced with empty vector (shFEV+vector) or ectopically expressed ITGA4 (shFEV+ITGA4) over a period of 7 days. **(B)** Frequency of cells in G1 phase in the NC, shFEV+vector and shFEV+ITGA4 groups. **(C)** Engraftment of human cells in BM, spleen and liver of mice that received MV4-11 cells in the NC, shFEV+vector and shFEV+ITGA4 groups after 15 days (n=3). **(D)** HE-stained sections (size bars = 50 μm) in BM, spleen and liver of mice that received MV4-11 cells in the NC, shFEV+vector and shFEV+ITGA4 groups after 15 days. **(E)** Conserved core binding motif of FEV. **(F)** Schematic of *ITGA4* promoter. CP indicates the control sites without the FEV binding site. P1 and P2 indicate the regions with conserved FEV binding sites. **(G)** ChIP-PCR assay showed that FEV could bind to both two regions of *ITGA4* promoter. The results are representative of at least three independent experiments. *P < 0.05, **P < 0.01, and ***P < 0.001 (Student's t test). All data are presented as the mean ± SD.

Supplementary Figure 7. Blocking integrin α4 activity reduces the expansion, migration and adhesion abilities of MV4-11 cells. (A) Number of colonies formed by MV4-11 cells after IgG4 or NZM treatment after 14 days. **(B)** Frequency of migrated MV4-11 cells after 2 days of IgG4 or NZM treatment. **(C)** Average count of adherent MV4-11 cells after 2 days of IgG4 or NZM treatment per view. **(D)** CCK-8 assay of MV4-11 cells after IgG4 or NZM treatment over a period of 7 days. **(E)** Frequency of cells in G1 phase in MV4-11 cells after 2 days of IgG4 or NZM treatment. The results are representative of at least three independent experiments. *P < 0.05 and ***P < 0.001 (Student's t test). All data are presented as the mean ± SD.

Supplementary Figure 8. Blocking integrin α4 activity reduces the colony-forming, migration and adhesion abilities of blasts and LICs in primary and relapsed AML patients. (A) Number of colonies formed by blasts sorted from primary AML patients after IgG4 or NZM treatment after 14 days. **(B)** Frequency of migrated blasts from primary AML patients after 2 days of IgG4 or NZM treatment. **(C)** Average count of adherent blasts from primary AML patients after 2 days of IgG4 or NZM treatment per view. The results are representative of at least three independent experiments. *P < 0.05 and **P < 0.01 (paired t test). All data are presented as the mean ± SD.

Supplementary Figure 1

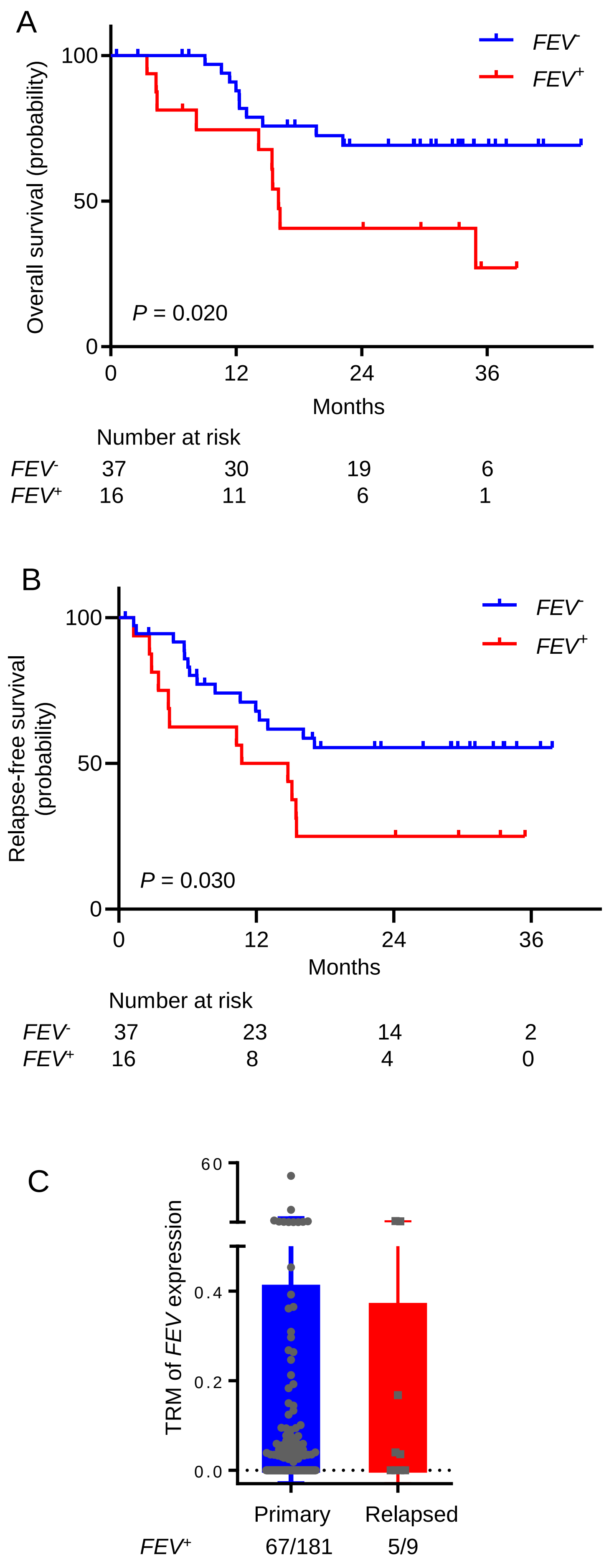

Supplementary Figure 2

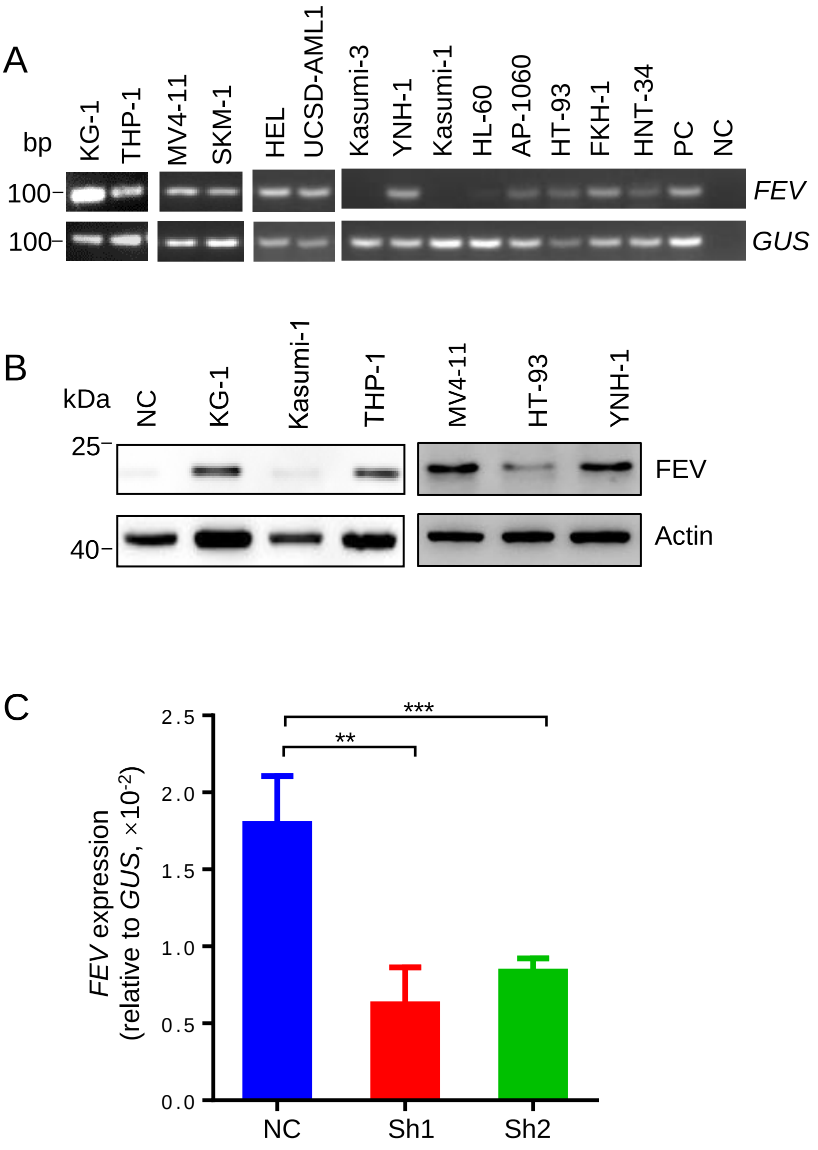

Supplementary Figure 3

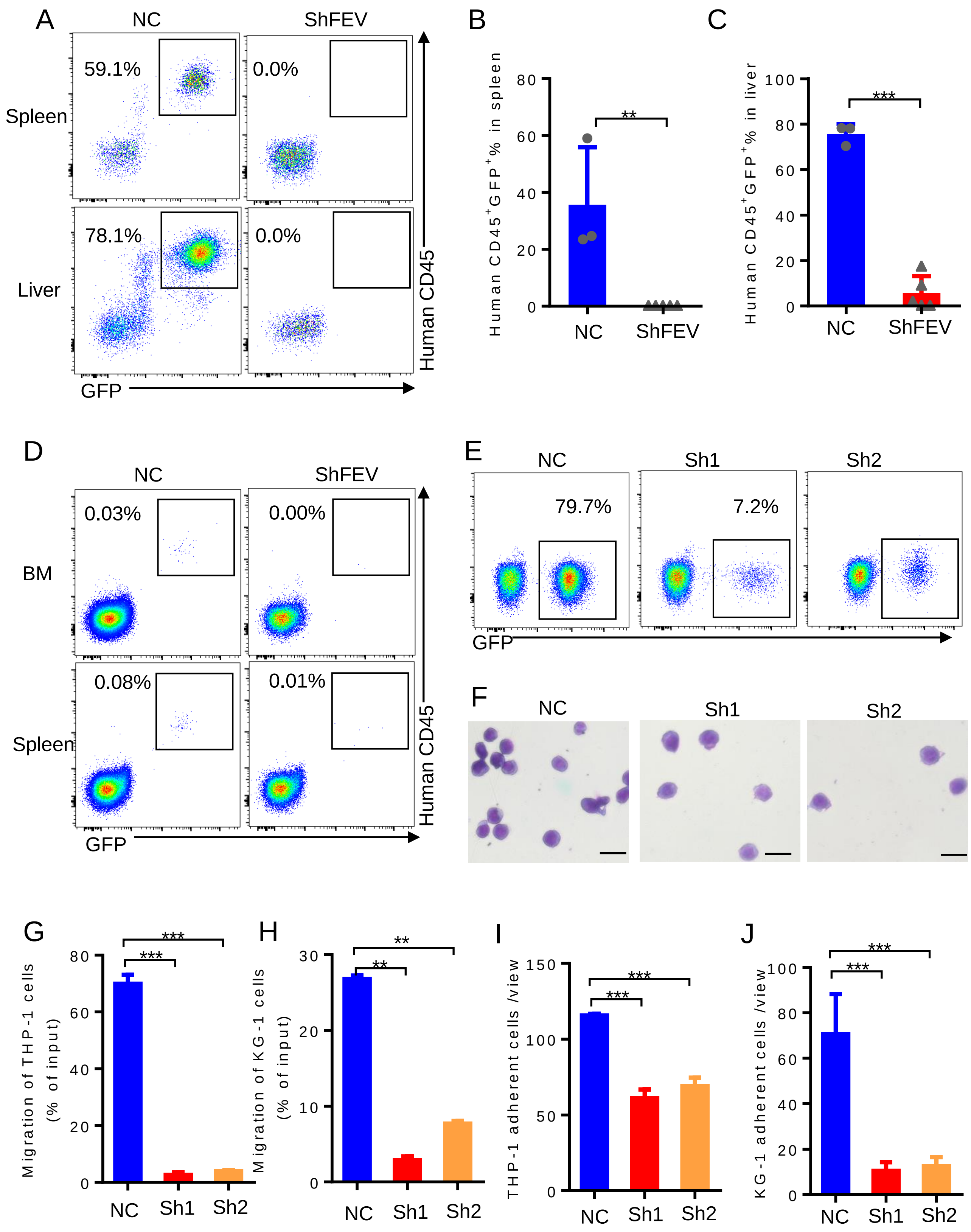

Supplementary Figure 4

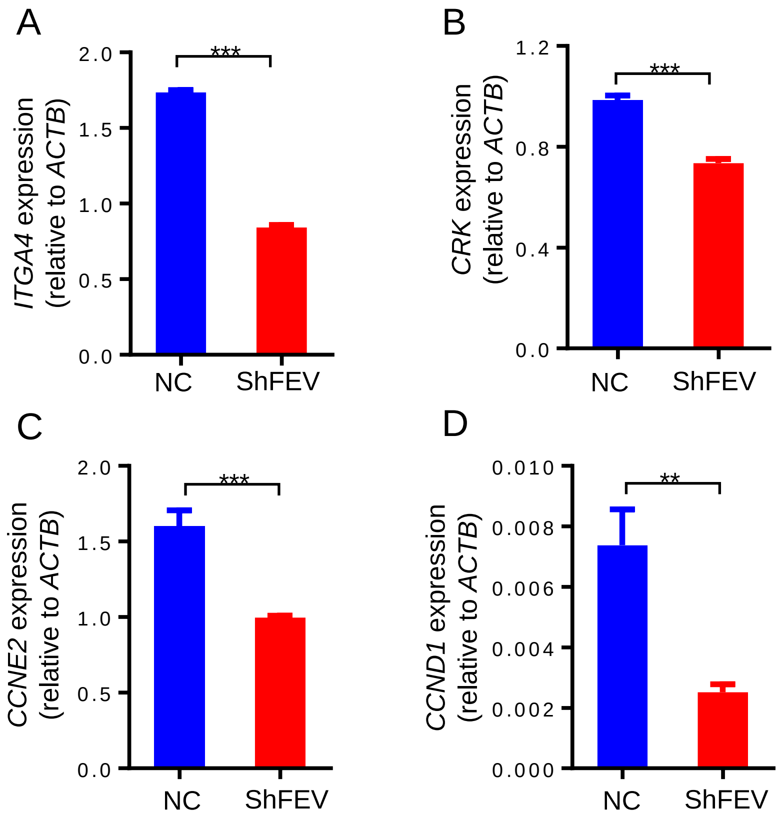

Supplementary Figure 5

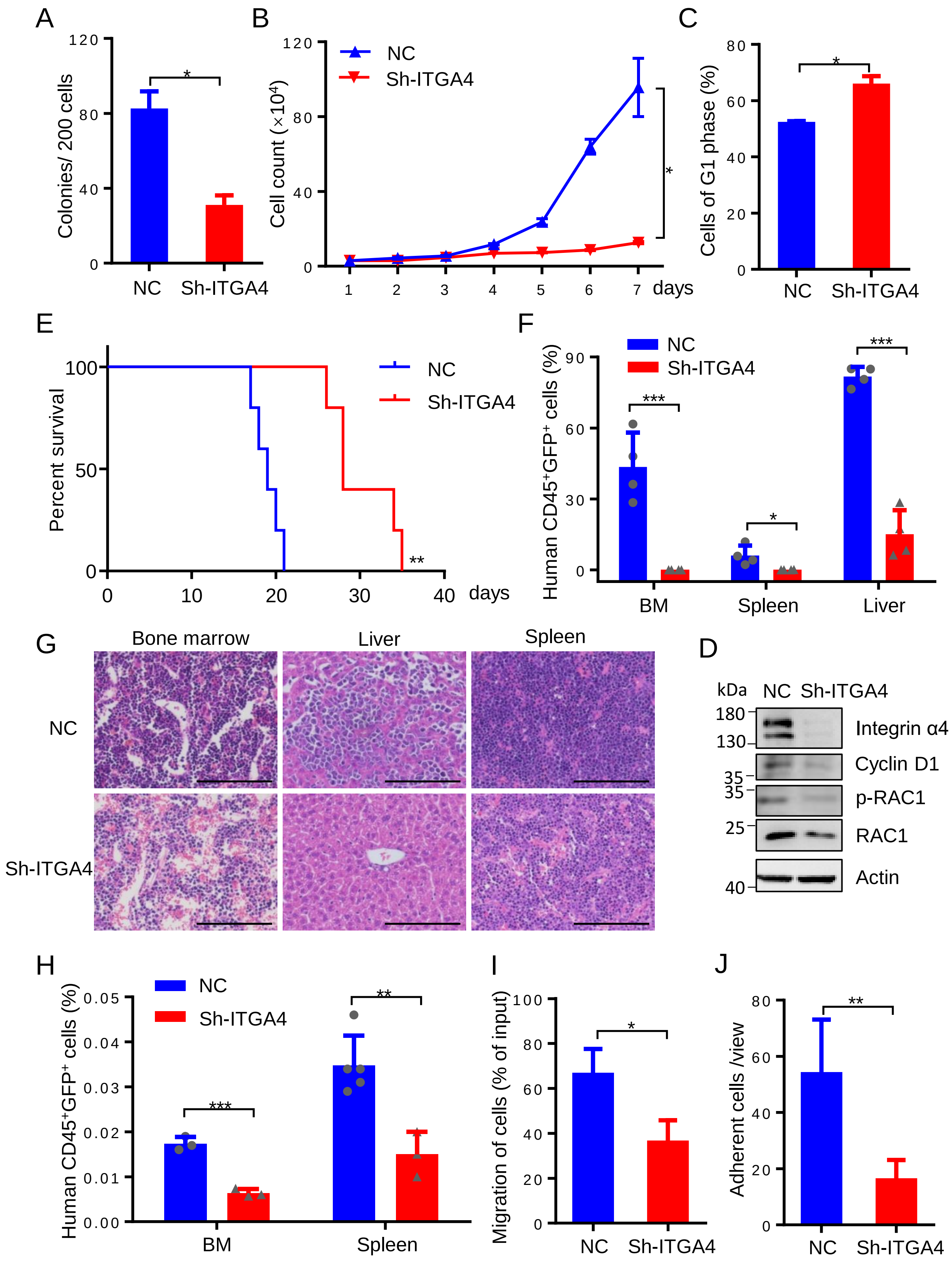

Supplementary Figure 6

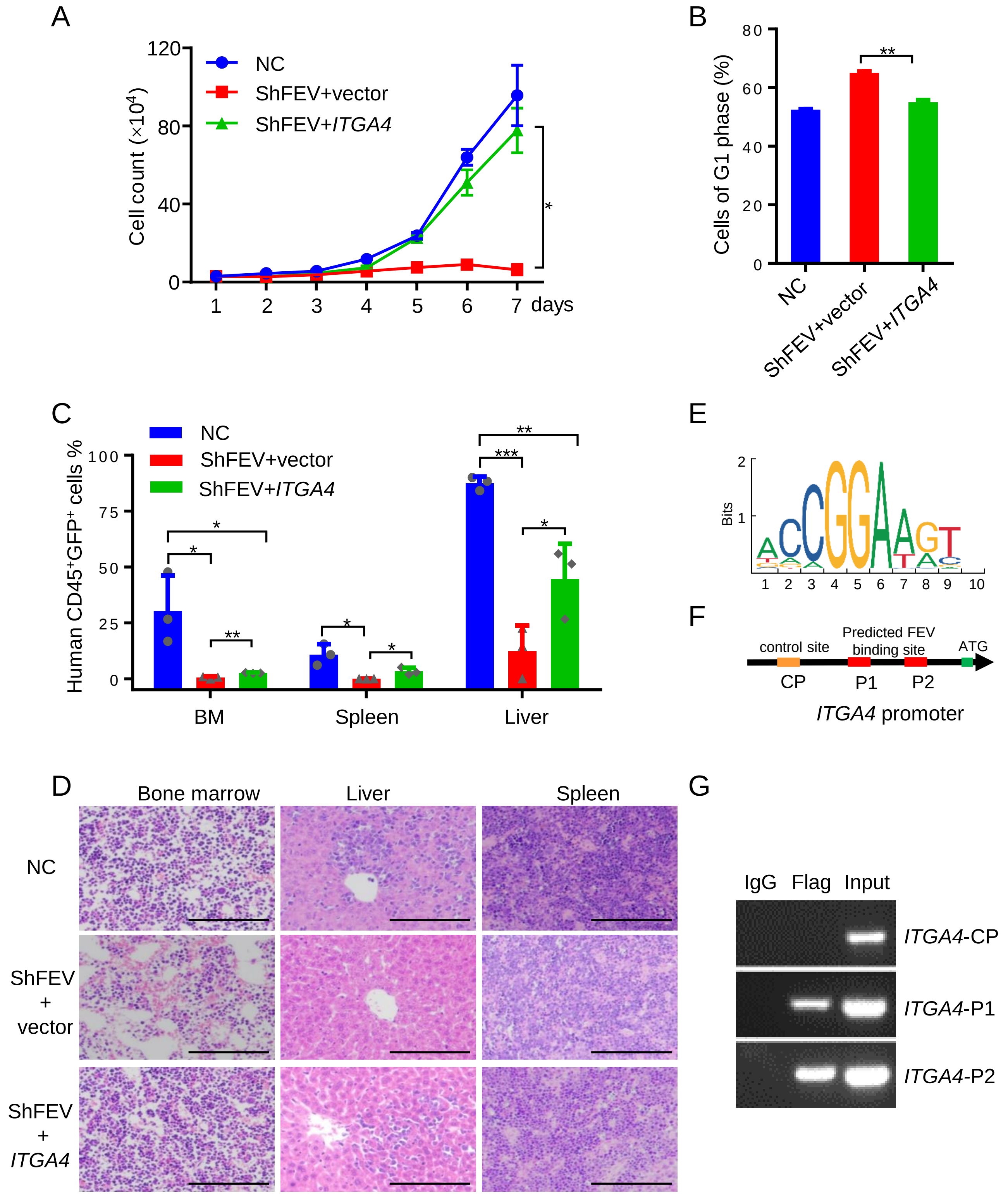

Supplementary Figure 7

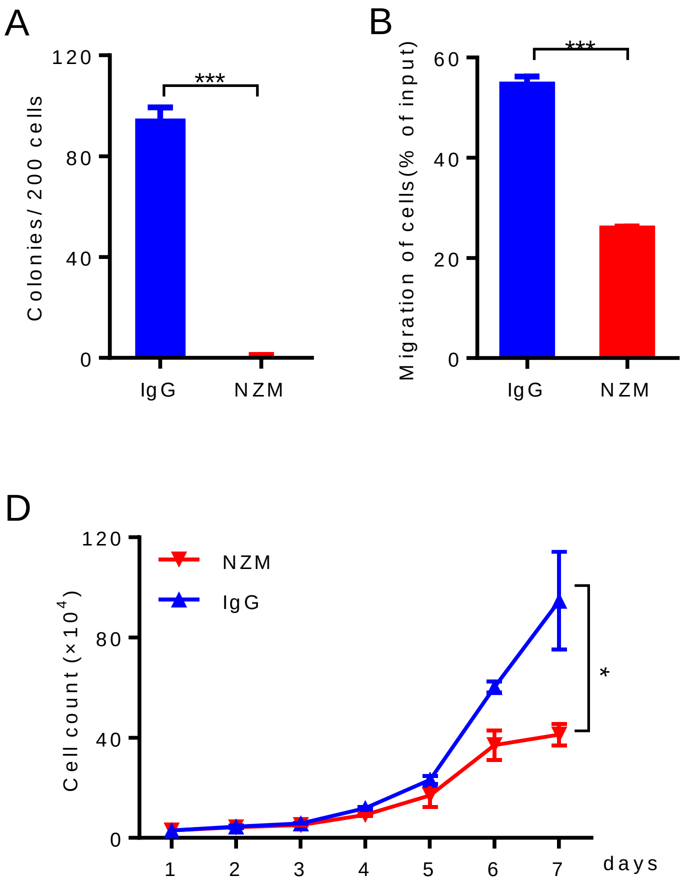

Supplementary Figure 8

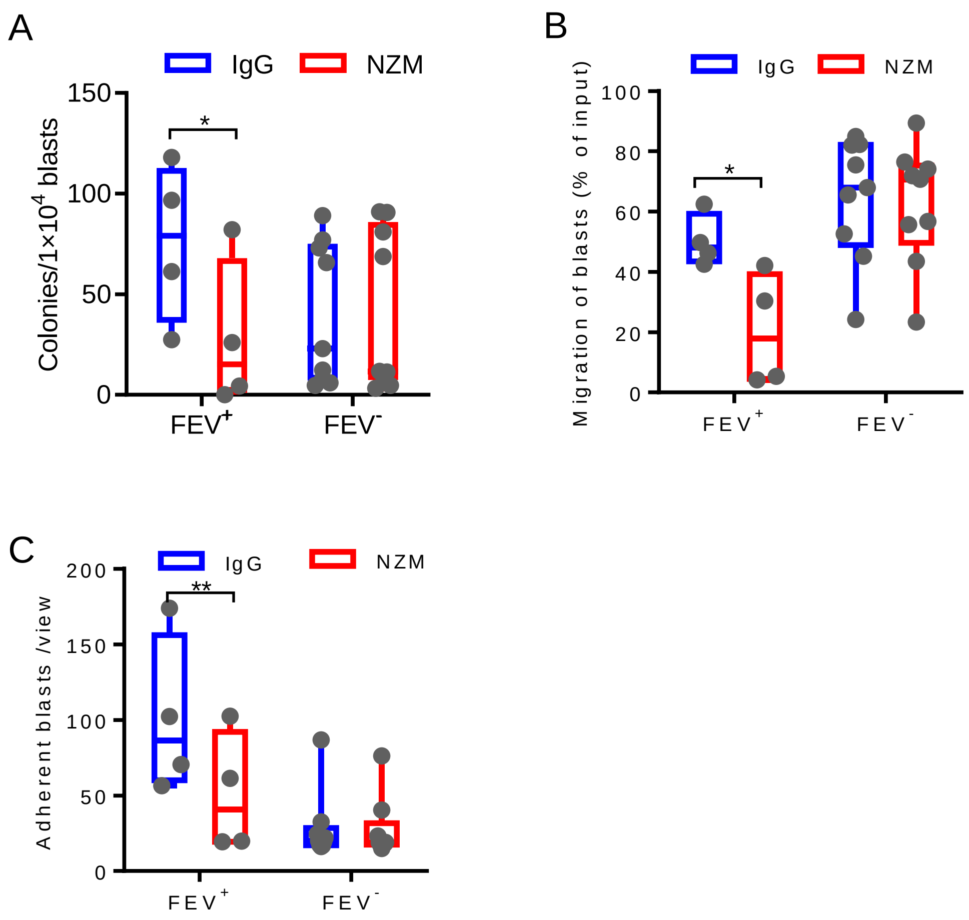

Supplementary Table 1. *FEV* expression in acute myeloid leukemia (AML) samples.

Sample ID	Patient ID	Gender	Age at diagnosis	Leukemia subtype	Stages of progression	*FEV* relative expression ($\times10^{-5}$)
1	P1	male	28	AML	primary	0.13
2	P1	male	28	AML	at relapse	0.42
3	P2	male	24	AML	primary	1.50
4	P2	male	24	AML	at relapse	1.26
5	P3	female	46	AML	primary	undetected
6	P3	female	46	AML	at relapse	0.26
7	P4	female	21	AML	primary	undetected
8	P4	female	21	AML	at relapse	0.52
9	P5	female	48	AML	primary	undetected
10	P5	female	48	AML	at relapse	0.12
11	P6	female	71	AML	primary	0.57
12	P6	female	71	AML	at relapse	0.91
13	P7	female	38	AML	primary	undetected
14	P7	female	38	AML	at relapse	0.88
15	P8	female	56	AML	primary	undetected
16	P8	female	56	AML	at relapse	44.42
17	P9	female	30	AML	primary	undetected
18	P9	female	30	AML	at relapse	0.08
19	P10	female	33	AML	primary	undetected
20	P10	female	33	AML	at relapse	0.62
21	P11	male	50	AML	primary	0.16
22	P11	male	50	AML	at relapse	0.09
23	P12	male	59	AML	primary	undetected
24	P12	male	59	AML	at relapse	0.15
25	P13	female	60	AML	primary	undetected
26	P14	female	46	AML	primary	undetected
27	P15	male	20	AML	primary	undetected
28	P16	male	33	AML	primary	undetected
29	P17	male	49	AML	primary	undetected
30	P18	female	55	AML	primary	undetected
31	P19	female	38	AML	primary	undetected
32	P20	male	33	AML	primary	undetected
33	P21	female	44	AML	primary	1.31
34	P22	male	41	AML	primary	undetected
35	P23	male	63	AML	primary	undetected
36	P24	female	58	AML	primary	undetected
37	P25	male	55	AML	primary	undetected
38	P26	female	57	AML	primary	undetected
39	P27	female	34	AML	primary	undetected
40	P28	male	39	AML	primary	undetected
41	P29	female	45	AML	primary	undetected
42	P30	male	32	AML	primary	undetected
43	P31	male	28	AML	primary	undetected
44	P32	female	48	AML	primary	undetected
45	P33	female	49	AML	primary	undetected
46	P34	male	33	AML	primary	undetected
47	P35	male	47	AML	primary	undetected
48	P36	female	31	AML	primary	undetected
49	P37	male	31	AML	primary	undetected
50	P38	male	25	AML	primary	undetected
51	P39	male	56	AML	primary	undetected
52	P40	male	27	AML	primary	undetected
53	P41	male	58	AML	primary	0.42
54	P42	female	64	AML	primary	undetected
55	P43	male	65	AML	primary	1.13
56	P44	female	42	AML	primary	0.68
57	P45	male	46	AML	primary	2.12
58	P46	male	34	AML	primary	0.33
59	P47	male	28	AML	primary	0.31

60	P48	male	19	AML	primary	0.61
61	P49	male	25	AML	primary	0.67
62	P50	male	39	AML	primary	0.51
63	P51	male	62	AML	primary	undetected
64	P52	female	35	AML	primary	0.47
65	P53	male	22	AML	primary	0.31
66	P54	female	46	AML	at relapse	0.15
67	P55	female	18	AML	at relapse	0.20
68	P56	male	33	AML	at relapse	2.55
69	P57	male	22	AML	at relapse	0.62

Supplementary Table 2. Clinical characteristics between *FEV*$^+$ and *FEV*$^-$ adult leukemic patients.

Characteristics	*FEV*$^-$ (n=37)	*FEV*$^+$ (n=16)	*P* Value
Age, years, median (range)	45 (20-64)	37 (19-71)	0.348
Sex, n (%)			0.130
Female	19 (51.4%)	4 (25.0%)	
Male	18 (48.6%)	12 (75.0%)	
WBC, $\times10^9$/L, median (range)	17.12 (0.71-362.20)	38.50 (2.70-307.00)	0.460
Hb, g/L, median (range)	88 (52, 125)	99 (65, 125)	0.054
PLT, $\times10^9$/L, median (range)	41 (6, 332)	64 (5, 594)	0.374
BM blast, %, median (range)	61.0 (20.0, 92.0)	69.4 (22.0, 90.0)	0.434
FAB subtype, n (%)			0.288
M1	6 (17.6%)	1 (6.7%)	
M2	8 (23.5%)	3 (20.0%)	
M4	11 (32.4%)	5 (33.3%)	
M5	6 (17.6%)	7 (46.7%)	
Not classified	5	1	
Risk category, n (%)			0.002
Favorable	18 (51.4%)	0 (0.0%)	
Intermediate	9 (25.7%)	8 (50.0%)	
Adverse	8 (22.9%)	8 (50.0%)	
Unknown	2	0	
Genetic mutation, n (%)			
CEBPA	13 (36.1%)	1 (6.3%)	0.041
DNMT3A	9 (25.0%)	5 (31.3%)	0.736
NPM1	9 (25.0%)	5 (31.3%)	0.736
FLT3-ITD	9 (25.0%)	8 (50.0%)	0.108
RUNX1	2 (5.6%)	2 (12.5%)	0.575
ASXL1	3 (8.3%)	0 (0.0%)	0.545
NRAS/KRAS	8 (22.2%)	3 (18.8%)	1.000
IDH1/2	5 (13.9%)	3 (18.8%)	0.685
TET2	7 (19.4%)	2 (12.5%)	0.706
GATA2	3 (8.3%)	1 (6.3%)	1.000
Cytogenetics, n (%)			
Normal	21 (58.3%)	13 (86.7%)	0.123
t(8;21)	5 (13.9%)	0 (0.0%)	0.307
inv(16)	3 (8.3%)	0 (0.0%)	0.545
t(6;9)	0 (0.0%)	1 (6.7%)	0.302
inv3	1 (2.8%)	0 (0.0%)	1.000
t(11;19)	1 (2.8%)	0 (0.0%)	1.000
Unknown	1	1	
Induction course, n (%)			<0.001
≤2	35 (94.6%)	2 (12.5%)	
>2	2 (5.4%)	14 (87.5%)	

Supplementary Table 3. Clinical characteristics of relapsed patients.

	Patient ID	P1	P2	P6	P11	P3	P4	P5	P7	P8	P9	P10	P12	P54	P55	P56	P57
**FEV**	Diagnosis	+	+	+	+	-	-	-	-	-	-	-	-	UK	UK	UK	UK
	Relapse	+	+	+	+	+	+	+	+	+	+	+	+	+	+	+	+
**Charactristic at diagnosis**	Sex	Male	Male	Female	Male	Female	Female	Female	Female	Female	Female	Female	Male	Female	Female	Male	Male
	Age	28	24	71	50	46	21	48	38	56	30	33	59	46	18	33	22
	FAB subtype	M5	M4	M5	M4	M4	M4	M5	M4	Not classified	M1	M2	M2	M1	M5	M2	M4b
	Risk category	Adverse	Intermediate	Intermediate	Intermediate	Adverse	Intermediate	Adverse	Intermediate	Intermediate	Adverse	Favorable	Adverse	Favorable	Adverse	Favorable	Adverse
	WBC ($\times 10^9$/L)	41.00	86.73	15.13	51.00	62.77	63.30	37.14	73.91	1.65	108.49	2.60	15.16	5.42	7.66	22.71	76.24
	Hb (g/L)	69	93	99	125	95	72	73	93	85	95	109	104	70	62	96	110
	PLT ($\times 10^9$/L)	34	23	41	84	332	137	54	130	24	80	71	6	30	34	30	92
	BM blast (%)	67.0	39.5	79.0	90.0	59.0	32.5	74.0	61.0	50.0	92.0	72.0	20.0	31.0	72.8	66.5	87.4
	Genetic mutation	FLT3-ITD, RUNX1	DNMT3A, FLT3-ITD, FLT3-TKD, NPM1	IDH1, FLT3, NPM1, NOTCH1	-	DNMT3A, IDH2, NRAS, FLT3-ITD, PTPN1	NPM1, FLT3, DNMT3A	FLT3-ITD, TET2, GATA2, ASXL1	FLT3-ITD, DNMT3A, NPM1	NMT3A, ARID1B	FLT3-ITD, CEBPA, NOTCH2, SETD2	TET2	ASXL1, CEBPA, IDH2, EP300	CEBPA, WT1, CSF3R	FLT3-ITD	FLT3-ITD, WT1, C-KIT	FLT3-ITD, RUNX1
	Cytogenetics	-	-	-	add(10)(p11.2), add(11)(q13)	-	-	-	-	-	-	t(8; 21)(q22, q22)	-	-	Trisomy 8	t(8, 21)(q22, q22)	-
	Induction course	>2	>2	>2	1	1	2	2	2	2	2	1	2	1	1	>2	1
	CR duration (months)	0.5	1.2	13.2	2.3	5.9	3.1	0.7	4.7	1.3	0.4	5.8	3.3	9.5	0.7	10.5	10.7

**Charactristic at relapse**	WBC (× 10^9/L)	3.32	2.24	4.17	4.13	1.06	4.64	12.88	2.92	7.91	7.12	3.24	1.79	1.2	1.04	17.26	26.97
	Hb (g/L)	124	77	97	113	88	102	114	85	69	102	77	110	92	52	109	74
	PLT (×10^9/L)	212	106	34	175	26	167	422	96	30	26	168	22	106	12	9	71
	BM blast (%)	22	66.5	83.5	16.0	17	11.5	39.0	19.0	54.0	21.5	1.0	31.5	36.0	47.0	68.5	79.0
	Genetic mutation	UK	FLT3-ITD, DNMT3A	CEBPA, IDH1, NPM1, FLT3-ITD	-	IDH2, DNMT3A, FLT3-ITD	FLT3-ITD, DNMT3A, NPM1	FLT3-ITD	-	DNMT3A	NOTCH1, FLT3-ITD	UK	ASXL1, CEBPA, IDH2	UK	FLT3-ITD	FLT3-ITD	FLT3-ITD, CEBPA, WT1
	Clinical outcome at relapse	Relapsed at +2.7m; alive	Relapsed at +1.3m and +6.7m; died of relapse at +8.2m	Relapsed at +15.1m and +28m; died of relapse at +34.9m	Relapsed at +2.9m; alive	Relapsed at +6.8m and +11.2m; died of relapse at +12.3m	Relapsed at +5.7m; alive	Relapsed at +1.5m; alive	Relapsed at +6m; died of relapse at +14.5m	Relapsed at +4.8m and +7.3m; died of relapse at +9m	Relapsed at +1.3m; alive	Relapsed at +6.2m; alive	Relapsed at +5.7m and +15.9m; died of relapse at +19.7m	Relapsed at +10.6m; alive	Relapsed at +1m; alive	Relapsed at +11.4m; died of relapse at +22.3m	Relapsed at +12.6m; died of relapse at +14.5m

Supplementary Table 4. Primers for RT-qPCR

Genes	Forward primer sequence (5′ to 3′)	Reverse primer sequence (5′ to 3′)
FEV	TTCAGAAAGGCAGCGGACAGA	GAGCTTGAACTCGCCGTGAC
ACTB	CATCCTCACCCTGAAGTACCC	AGCCTGGATAGCAACGTACATG
GUS	GACACGCTAGAGCATGAGGG	GGGTGAGTGTGTTGTTGATGG
ITGA4	GCTTCTCAGATCTGCTCGTG	GTCACTTCCAACGAGGTTTG
CRK	GGAGACATCTTGAGAATCCGGG	ACGTAAGGGACTGGAATCATCC
CCND1	GCTGCGAAGTGGAAACCATC	CCTCCTTCTGCACACATTTGAA
CCNE2	TCAAGACGAAGTAGCCGTTTAC	TGACATCCTGGGTAGTTTTCCTC

Supplementary Table 5. Pathway analysis of the differentially expressed genes in FEV knockdown cells.

Pathway	*P* -Value	Corrected *P* -Value
Metabolic pathways	7.65E-19	2.4E-16
Pathways in cancer	1.12E-13	1.75E-11
Phagosome	2.21E-11	2.31E-09
Tuberculosis	2.77E-10	2.17E-08
Antigen processing and presentation	1.63E-09	9.71E-08
Cell adhesion molecules (CAMs)	1.85E-09	9.71E-08
Hematopoietic cell lineage	3.44E-09	0.000000148
PI3K-Akt signaling pathway	3.77E-09	0.000000148
Human T-cell leukemia virus 1 infection	2.75E-08	0.000000959
Toxoplasmosis	4.35E-08	0.00000137
Human cytomegalovirus infection	4.98E-08	0.00000142
Cytokine-cytokine receptor interaction	0.000000057	0.00000149
Osteoclast differentiation	0.000000095	0.00000229
Epstein-Barr virus infection	0.000000115	0.00000258
Kaposi sarcoma-associated herpesvirus infection	0.00000022	0.00000432
Transcriptional misregulation in cancer	0.00000022	0.00000432
Type I diabetes mellitus	0.000000399	0.00000738
Viral myocarditis	0.000000586	0.0000102
Allograft rejection	0.000000651	0.0000108
Leishmaniasis	0.00000155	0.0000244
Endocytosis	0.00000196	0.0000294
Ribosome biogenesis in eukaryotes	0.00000247	0.0000353
Apoptosis	0.00000271	0.000037
Rheumatoid arthritis	0.0000049	0.0000641
Graft-versus-host disease	0.0000067	0.0000842
Chemokine signaling pathway	0.00000725	0.0000876
ECM-receptor interaction	0.00000896	0.00010423
Staphylococcus aureus infection	0.00000962	0.00010791
Autoimmune thyroid disease	0.0000142	0.000153815
p53 signaling pathway	0.0000174	0.000181691
NOD-like receptor signaling pathway	0.0000186	0.000188115
Intestinal immune network for IgA production	0.0000306	0.000299916
Small cell lung cancer	0.0000716	0.000681008
Cellular senescence	0.000076	0.000701478
Legionellosis	0.0000805	0.000721807
Human papillomavirus infection	0.000105607	0.000921129
Insulin resistance	0.000114151	0.000968741
